# Clinical Features of Severe Ovarian Hyperstimulation Syndrome with Hydrothorax

**DOI:** 10.3390/jcm12196210

**Published:** 2023-09-26

**Authors:** Xiaowei Ma, Jingwen Yin, Rui Yang, Shuo Yang, Jia Li, Yang Wang, Rong Li

**Affiliations:** 1 Center for Reproductive Medicine, Department of Obstetrics and Gynecology, Peking University Third Hospital, Beijing 100191, China; 13691009139@163.com (X.M.); yjw010203@163.com (J.Y.); yrjeff@126.com (R.Y.); yangshuo@263.net (S.Y.); jj_apple_jj@163.com (J.L.); 2 National Clinical Research Center for Obstetrics and Gynecology, Peking University Third Hospital, Beijing 100191, China; 3 Key Laboratory of Assisted Reproduction, Peking University, Ministry of Education, Beijing 100191, China; 4 Beijing Key Laboratory of Reproductive Endocrinology and Assisted Reproductive Technology, Beijing 100191, China

**Keywords:** OHSS, hydrothorax, clinical features, IVF, live birth

## Abstract

Problem: Does the presence of hydrothorax suggest that severe ovarian hyperstimulation syndrome (OHSS) is associated with more severe conditions and worse pregnancy outcomes? Method of study: The clinical data for 868 hospital patients with severe OHSS following IVF-ET at Peking University Third Hospital between 1 January 2016 and 21 July 2021 were retrospectively analysed. The patients were divided into two groups, the ascites alone group (n = 417) and the ascites combined with hydrothorax group (n = 451), to investigate the clinical features and pregnancy outcomes of patients with severe ovarian hyperstimulation syndrome (OHSS) combined with hydrothorax plus ascites. Results: The clinical data for 868 hospital patients with severe OHSS following IVF-ET were included. A total of 51.96% of patients with severe OHSS had hydrothorax plus ascites, mainly bilateral and moderate hydrothorax. Most cases with hydrothorax could be monitored and observed, and only 2.66% of the cases required thoracentesis and pleural drainage. Clinically, the time to visit due to worsening symptoms was longer; the hospital stay was shorter; and the OHSS-related laboratory tests, such as white blood cells (WBC), haematocrit (HCT), and ovarian diameter, were less severe in the ascites combined with hydrothorax group than in the ascites alone group. For live-birth outcomes of IVF-ET, the presence and the volume of hydrothorax were not independent risk factors, while the late onset of OHSS (odds ratio [OR]: 0.857 95% confidence interval [CI]: 0.795, 0.925) and a history of foetal reduction (OR: 13.796 95% CI: 1.808, 105.288) were independent protective factors for live birth. Conclusions: Patients with severe OHSS combined with hydrothorax plus ascites have less severe clinical manifestations and laboratory tests than those with ascites alone. The presence and the volume of hydrothorax are unrelated to live-birth outcomes following in vitro fertilization and embryo transfer (IVF-ET).

## 1. Introduction

Ovarian hyperstimulation syndrome (OHSS) is the most important iatrogenic complication during the process of controlled ovulation hyperstimulation (COH). The overall incidence is 3–10% in all assisted reproductive treatment (ART) cycles. The incidence of severe OHSS is 0.1–2.0% with a mortality rate of 1:45,000–1:50,000 [1]. OHSS is characterized by ovarian enlargement, ascites, hydrothorax, haemoconcentration, and oliguria. In severe cases, OHSS can cause thrombosis, acute respiratory distress syndrome, renal failure, and even death. OHSS may be mild, moderate, severe, or critical according to the severity of clinical manifestations and laboratory tests. Hydrothorax is one manifestation of severe and critical OHSS [2] with an incidence of 23–29%. Hydrothorax is more common on the right side due to the anatomy of the right diaphragm [3,4,5]. For patients with severe OHSS, it is unclear whether the presence of pleural effusion indicates more severe conditions or worse pregnancy outcomes. Our research is designed to answer this question. Also, the clinical data and pathogenesis of patients with severe OHSS following COH were analysed to investigate the impact of hydrothorax on severe OHSS as a reference for clinical decisions. 

## 2. Materials and Methods 

### 2.1. Data Collection 

The clinical data for 868 hospital patients with severe OHSS following COH and IVF-ET at Peking University Third Hospital between 1 January 2016 and 21 July 2021 were retrospectively analysed. The patients were required to have undergone ultrasound assessment of hydrothorax and ascites to be eligible. The exclusion criteria were as follows: (1) OHSS caused by ART other than IVF-ET; (2) spontaneous OHSS; (3) mild or moderate OHSS; and (4) no assessment of hydrothorax or ascites. The 868 patients with severe OHSS were divided into two groups: the ascites alone group (n = 417) and the ascites combined with hydrothorax group (n = 451). Demographics and information about IVF-ET, pregnancy outcomes, and the laboratory tests during the onset and treatment of OHSS were collected to analyse the clinical features and pregnancy outcomes of the patients in the ascites combined with hydrothorax group. 

### 2.2. Methods

#### 2.2.1. COH, Oocyte Retrieval, and Fresh ET

Ovarian reserve was evaluated based on the patient’s age and baseline sex hormones, and then the ovulation hyperstimulation protocol and the initial dose of gonadotropin (Gn) were determined. The growth of follicles was monitored by ultrasound, and the dosage of Gn was adjusted accordingly. The timing of the trigger was determined based on the follicle diameter and the level of serum sex hormones on the night when the diameter of two follicles was ≥18 mm. Oocytes were retrieved within 34 to 36 h under the guidance of vaginal ultrasound, followed by routine IVF or intracytoplasmic sperm injection (ICSI). Three to five days later, one to two transferable embryos were transferred. An increased risk of OHSS was considered if (1) oestrogen 2 (E2) ≥ 15,000 pmol/L on the trigger day, (2) the number of oocytes retrieved ≥20, or (3) E2 ≥ 10,000 pmol/L on the trigger day, and the number of oocytes retrieved ≥15. In this case, fresh ET was terminated, and whole-embryo cryopreservation was performed to reduce the risk of OHSS. 

#### 2.2.2. Luteal Phase Support and Pregnancy Monitoring

For fresh ET, all recipients were given vaginal progesterone gel alone or combined with oral dydrogesterone (20 mg/d) for luteal phase support from the day of oocyte retrieval. Serum human chorionic gonadotropin (hCG) was determined 10 to 14 days after transfer, and clinical pregnancy was considered if ultrasound on day 30 showed foetal germ cells and embryonic heart pulsation. The patients were followed up with by phone to monitor the mother and the unborn baby and record any pregnancy complications, gestational week at delivery, and delivery method. 

#### 2.2.3. Diagnostic Criteria for Severe OHSS

Severe OHSS was diagnosed according to the 2016 American Society for Reproductive Medicine (ASRM) Prevention and Treatment of Moderate and Severe Ovarian Hyperstimulation Syndrome: A Guideline [2]. 

#### 2.2.4. Definition and Measurement of Hydrothorax and Ascites

Ascites was estimated by ultrasound based on the scope and depth of effusion in the supine position. Mild ascites was defined as 1 to 2 areas with a maximum effusion depth < 2 cm, moderate ascites was defined as 2 to 5 areas with a maximum effusion depth ≤ 5 cm, and massive ascites was defined as more than 5 areas with a maximum effusion depth > 5 cm. Hydrothorax (mL) was estimated by ultrasound using the following formula: 90 × maximum effusion height (cm); less than 500 mL indicated mild hydrothorax, 500–1000 mL indicated moderate hydrothorax, and >1000 mL indicated massive hydrothorax. 

#### 2.2.5. Post-Admission Monitoring and Treatments

(1) Twenty-four-hour intake and output, abdominal circumference, weight and vital signs, and abdominal and pulmonary signs were monitored daily; (2) blood test, urine analysis, and haemoconcentration were monitored every day or every other day; (3) blood chemistry (including electrolytes, liver and kidney function, albumin, total protein) was monitored every 3 to 4 days or as needed. All patients received symptomatic and supportive treatments, such as diet and lifestyle guidance, hydration with crystalloid and colloid fluids, hepatoprotection, anticoagulation, and albumin infusion, to correct hypoalbuminaemia. Paracentesis and thoracentesis were performed as needed for peritoneal or pleural drainage to relieve symptoms. Indications for patients who needed thoracentesis in this study: those who could not lie down at night, had chest tightness and heavy breathing, ultrasound showed a large amount of pleural effusion, and those with ascites whose symptoms were not relieved with abdominal puncture and drainage of ascites.

#### 2.2.6. Outcome Measures and Definitions

Live birth was defined as live birth at or after gestational week 28. The live birth rate (LBR) was calculated as the number of women who achieved live birth divided by the number of women who accepted fresh ET. Full-term birth was defined as birth between gestational weeks 37 and 42. The full-term birth rate was calculated as the number of women who achieved full-term birth divided by the number of live births. Miscarriage was defined as the loss of a clinical pregnancy before 28 weeks’ gestation and was classified as early miscarriage (pregnancy loss before 12 weeks) and mid-term miscarriage (loss at 12–28 weeks). The miscarriage rate (MR) was calculated as the number of miscarriages divided by the number of women who accepted fresh ET. 

### 2.3. Statistical Analysis

SPSS v25.0 was used for statistical analysis. Normally distributed measurement data are expressed as $\bar{x}$ ± s and analysed with the independent sample *t*-test; non-normally distributed measurement data are expressed as the median (min, max) and analysed with the rank sum test. Count data are expressed as percentages and analysed with the χ^2^ test or Fisher’s exact test. Multivariate logistic regression was used to analyse relevant factors. *p* < 0.05 was considered statistically significant. 

## 3. Results

### 3.1. Basic Characteristics 

**Figure 1:**
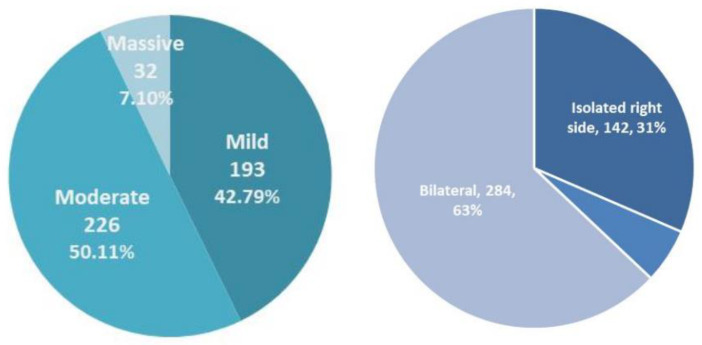
The severity and distribution of hydrothorax.

For the 868 patients with severe OHSS, the average age was 30.73 ± 3.60 years, and the average hospital stay was 3.44 ± 3.00 days. All patients had ascites, and 68.43% (594/868) had massive ascites. The incidence of hydrothorax was 51.96% (451/868). The severity and distribution of hydrothorax are shown in Figure 1. Moreover, 43.32% (376/868) of the patients underwent paracentesis and peritoneal drainage. The average volume of peritoneal drainage was 0.51 L (0.09, 23.80) over 2.44 days (1, 10); 2.66% (12/451) of the patients underwent thoracentesis and pleural drainage. The average volume of pleural drainage was 0.81 L (0.30, 1). A total of 437 cases of severe OHSS occurred after fresh ET. The overall LBR was 83.75% (366/437), the miscarriage rate was 10.30% (45/437), and the full-term birth rate was 76.78% (281/366).

### 3.2. Demographics and Baseline Characteristics 

Age, body mass index (BMI), and baseline E2 were significantly higher in the ascites combined with hydrothorax group than in the ascites alone group (*p* < 0.05). In contrast, E2 and progesterone (P) on the trigger day and the number of oocytes retrieved were significantly lower in the ascites combined with hydrothorax group than in the ascites alone group (*p* < 0.05). No significant between-group differences were observed in antral follicle count (AFC), male semen quality, IVF indications, ovulation hyperstimulation protocol, the dosage of Gn, or the trigger medication (*p* > 0.05). Details are presented in Table 1.

### 3.3. Clinical Features and Treatment of OHSS

For OHSS, the average time to symptomatic visit was 11.96 days after the trigger in the ascites combined with hydrothorax group, which was significantly later than that in the ascites alone group (10.57 days; *p* < 0.001). Moreover, the mean hospital stay was shorter (*p* < 0.001), and laboratory tests that reflected the severity of OHSS (such as WBC, HCT, ovarian diameter, TP, and ALB) were significantly less severe (*p* < 0.001) in the ascites combined with hydrothorax group. In the ascites alone group, abdominal symptoms were more severe with abdominal pain in 11.27% of the patients, massive ascites in 79.86% of the patients, and peritoneal drainage in 50.84% of the patients (average volume of peritoneal drainage: 2494.19 ± 3501.80 mL). All these indicators were significantly worse than those in the ascites combined with hydrothorax group (*p* < 0.05). However, in the ascites combined with hydrothorax group, chest symptoms were more severe, with chest tightness and shortness of breath in 57.43% of the patients, a significantly higher proportion than that in the ascites alone group (16.79%; *p* < 0.001). Details are presented in Table 2. 

### 3.4. IVF-Assisted Pregnancy Outcomes

The LBR was 87.67% in the ascites combined with hydrothorax group, which was significantly higher than that in the ascites alone group (79.52%; *p* = 0.021), while the mid-term miscarriage rate and the overall miscarriage rate were significantly lower (*p* < 0.05). No significant differences were observed in the foetal reduction rate, full-term birth rate, gestational week at delivery, or pregnancy complications (*p* > 0.05). Details are presented in Table 3. 

### 3.5. Multivariate Analysis of Factors for Live-Birth Outcomes in Patients with Severe OHSS

All 437 patients with severe OHSS following fresh ET were included in the univariate analysis to identify the factors for live birth (see Supplementary Table S1). The results indicated that the presence and volume of hydrothorax were significantly related to live birth (*p* < 0.05). Further logistic analysis that incorporated age, BMI, the number of embryos transferred, the type of embryos transferred, and variables with *p* < 0.1 in univariate analysis (previous IVF failure, total number of oocytes retrieved, time of symptom onset, abdominal symptoms, history of foetal reduction, Gn dosage, WBC) showed that the presence and the volume of hydrothorax were not independent risk factors for live birth (Table 4). While the late onset of OHSS and a history of foetal reduction were independent protective factors, a large number of oocytes retrieved, previous IVF failure, and cleavage-stage ET were independent risk factors for live birth (Table 4). 

## 4. Discussion

### 4.1. Clinical Manifestations and Pathophysiological Mechanisms of OHSS

As a complication of exogenous Gn-induced ovulation hyperstimulation, OHSS is characterized by ovary enlargement, increased vascular permeability, fluid in the third body cavity, and related pathophysiological processes. It is life-threatening in severe cases and is the most common and potentially dangerous complication of ART. Ascites is the first and most common symptom of OHSS, and 99% of patients with severe OHSS have ascites [6]. As the volume of ascites increases, the compliance of the abdominal wall decreases, and the venous drainage of the abdomen is impaired. Patients gradually develop bloating, abdominal pain, and high intra-abdominal pressure (IAP), as well as renal, gastrointestinal, liver, and respiratory disorders [7]. Oliguria is an early symptom of IAP, followed by nausea and vomiting caused by intestinal wall oedema and paralytic intestinal obstruction, decreased liver perfusion, elevated liver enzymes due to local ischemic-hypoxic injury, and hyponatremia and electrolyte disorder due to acute renal failure. Leucocytosis and elevated HCT and D-dimer indicate haemoconcentration and hypercoagulation. Moreover, increased pressure on pelvic vessels due to a high IAP, pregnancy status, or elevated E2 further increases the risk of thrombosis [8,9]. In this study, all patients with severe OHSS had varying degrees of ascites. Most had massive ascites (80%) and ascites alone. Compared with the ascites combined with hydrothorax group, the symptoms appeared earlier, abdominal pain and haemoconcentration were more severe, the ovaries were larger, and the hospital stay was longer. The shunting of peritoneal fluid to the pleural cavity may reduce IAP, improve blood circulation to pelvic and abdominal organs, improve venous drainage, and reduce liver, kidney, and gastrointestinal injury, thereby improving the clinical manifestations and laboratory tests of OHSS. 

### 4.2. OHSS and Hydrothorax

The presence of hydrothorax is a sign of severe OHSS. Studies have found that 23–29% of patients with severe or critical OHSS have hydrothorax (right side alone: 52–53%, bilateral: 27–29%, left side alone: 18–21%) [3,4,5]. Unlike previous studies, we performed noninvasive chest ultrasonography on all patients with severe OHSS and found that 52% of the patients had hydrothorax (bilateral: >60%; mild to moderate: 93%; massive: 7%). This discrepancy may be related to the small sample sizes and underestimation of the incidence of hydrothorax in previous studies; in most cases in previous studies, hydrothorax resolved on its own, and the patients did not undergo follow-up imaging [10]. 

The mechanism of hydrothorax in OHSS patients is unknown but may involve the following factors: (1) Cytokines and vasoactive substances secreted by the enlarged ovary following ovulation hyperstimulation lead to increased capillary permeability, and a large volume of body fluid extravasates into the third space. Vasoactive substances act selectively on the pleura, aggravating hydrothorax. Vascular endothelial growth factor (VEGF) and interleukin-6 (IL-6) play important roles in this process [4]. As a potent endothelial cell mitogen and vascular endothelial growth factor, VEGF promotes vascular endothelial proliferation and migration, increases vascular permeability, and accelerates the development of new vessels. It is 1000 times more potent than histamine in increasing capillary permeability. The expression of VEGF is significantly increased in the granulosa cells, follicular fluid, and ascites of patients with OHSS [11,12]. As a key member of the cytokine network, IL-6 has a wide range of biological effects. IL-6 is expressed in granulosa cells and luteal cells in the ovary. The expression of IL-6 is significantly upregulated in the follicular fluid, ascites, serum, and pleural fluid of OHSS patients. In addition to increasing capillary permeability, IL-6 may cause leukocytosis and elevated acute-phase protein synthesis in the liver, leading to a highly inflammatory environment [12]. (2) Anatomical diaphragmatic defects: With a high IAP, peritoneal fluid may enter the pleural cavity through the pores of the diaphragm to cause hydrothorax. Laparotomy, caesarean section, and autopsy have revealed significant diaphragmatic defects in which the diaphragm is only covered by a thin membrane in some cases [13]; radioisotope scanning and ultrasound have also confirmed the presence of valve-shaped pores in the diaphragm. There are four types of diaphragms (I–IV) according to the size of the diaphragmatic hiatus: type I, no obvious defect; type II, blebs lying on the diaphragm; type III, broken defects (fenestrations) in the diaphragm; and type IV, multiple gaps in the diaphragm. Type II and type III are more common, and the one-way valve on the diaphragm allows the peritoneal fluid to enter the pleural cavity (one-way) during breathing. Even with mild ascites, peritoneal fluid can be pumped into the pleural cavity by the negative pressure in the pleural cavity [14]. The dome of the right diaphragm is higher, which leads to a stronger suction effect and may partially explain why hydrothorax is more common on the right side. (3) Diaphragmatic lymphatic drainage: The lymphatic network is well-developed on both sides of the diaphragm. Peritoneal fluid can be absorbed into the diaphragmatic lymphatic vessels and transfer and accumulate in the pleural cavity. The lymphatic network is more developed on the right side, another possible reason why hydrothorax is more common on the right side [5]. 

### 4.3. The Impact of OHSS on the Outcomes of IVF-ET Assisted Pregnancy

A meta-analysis in 2021 revealed that the overall incidence of adverse pregnancy outcomes is 8.8 times higher in OHSS patients than in non-OHSS patients [15], and whether severe OHSS increases the risk of miscarriage is controversial. Studies have found that the overall miscarriage rate is 12.2–29.8% in OHSS patients [16,17,18]. The discrepancy across studies is related to the heterogeneity of the study population and the severity of OHSS. In this study, the overall miscarriage rate was 10.3%, consistent with previous findings. The miscarriage rate, especially the mid-term miscarriage rate, was significantly higher in the ascites alone group than in the ascites combined with hydrothorax group. The mechanism of miscarriage in OHSS patients is unknown. The following factors may be involved: (1) excessive serum E2 and cytokines. Excessive E2 from ovulation hyperstimulation to peri-implantation and early embryonic development may lead to increased miscarriage rates by affecting early embryonic development and endometrial receptivity [19]. Moreover, excessive cytokines, renin, angiotensin, and prostaglandin play a synergistic role. (2) Microthrombosis: Up to 86.7% of patients with severe OHSS have positive thrombus markers, such as D-dimer, and microthrombosis at the implantation site increases the likelihood of miscarriage [20]. (3) Hemodynamic instability, hemoconcentration, electrolyte disorder, and liver and kidney dysfunction are potential risk factors. Compared with the ascites combined with hydrothorax group, abdominal symptoms and ascites were more severe, and the ovaries were larger and occupied more pelvic and abdominal space in the ascites alone group, leading to a high IAP and reduced blood circulation to the uterus and placenta, which was further aggravated by more severe hemoconcentration. All these factors may significantly increase the risk of miscarriage. Early intervention and proactive treatment may help improve the pregnancy outcomes of patients with OHSS. 

Live birth is an important measure to evaluate the success rate of IVF. To date, few studies have investigated the live-birth outcomes across OHSS patients with different clinical features. With hydrothorax, the symptoms of chest tightness and shortness of breath or breathlessness can cause anxiety in patients and concerns about the adverse impact of potential hypoxemia on pregnancy. Most previous studies have been case reports of severe OHSS with idiopathic unilateral massive hydrothorax, and studies on the incidence and impact of hydrothorax on live-birth outcomes in the general OHSS population are scarce. In this study, hydrothorax data for more than 800 patients with severe OHSS were analysed. Univariate analysis revealed that the LBR was higher in the ascites combined with hydrothorax group. After balancing potential factors for live-birth outcomes, the results indicated that the presence and the volume of hydrothorax are unrelated to live-birth outcomes. Like routine IVF-assisted pregnancy, the number of embryos transferred, the type of embryos, and previous IVF failure were the primary factors for live births. In addition, foetal reduction for multiple gestation pregnancies played an important role in improving the live-birth outcomes of patients with severe OHSS and is an independent protective factor for live births in these patients. Therefore, active foetal reduction is advised for multiple gestation pregnancies in patients with severe OHSS to reduce the miscarriage rate and improve the LBR. Moreover, selective single embryo transfer reduces the risk of multiple gestation pregnancies and the risk of OHSS. 

Our study explores the impact of pleural effusion on severe OHSS and provides a reference for clinical decision-making. However, as a clinical retrospective study, its limitation is that there may be potential bias, but this is also due to the particularity of the patients included in this study.

## 5. Conclusions

More than 50% of patients with severe OHSS may have hydrothorax. The presence of hydrothorax does not suggest worsening conditions of severe OHSS; on the contrary, the shunting of peritoneal fluid to the pleural cavity may alleviate the IAP and ischemic-hypoxic injury of pelvic and abdominal organs, thereby improving the condition of OHSS. Patients with severe OHSS following IVF-assisted pregnancy are categorized into the high-risk pregnancy group. Once diagnosed, patients with severe OHSS should receive symptomatic and supportive treatments, such as hydration, anticoagulation, albumin infusion, and drainage of the third space fluid, to stabilize haemodynamics, improve hypoxemia, and relieve hypercoagulation, thereby reducing the miscarriage rate and improving the LBR. Active foetal reduction is advised for patients with multiple gestation pregnancies, and selective single ET may be considered for patients with an increased risk of OHSS. These measures reduce the risk of OHSS and effectively reduce pregnancy complications. 

## Figures and Tables

**Table 1:** Demographics and baseline characteristics of the OHSS participants received IVF-ET treatment with or without hydrothorax.

	Ascites Alone Group (n = 417)	Ascites Combined with Hydrothorax Group (n = 451)	*p*
Age	30.43 ± 3.45	30.99 ± 3.71	0.023
BMI (kg/m^2^)	22.02 ± 3.34	22.56 ± 3.17	0.015
Duration of infertility (years)	3.59 ± 2.48	3.38 ± 2.53	0.217
Gravidity (times)	0.54 ± 0.97	0.51 ± 0.81	0.545
Delivery (times)	0.04 ± 0.22	0.03 ± 0.17	0.297
Basal FSH (MIU/mL)	5.05 ± 2.33	5.31 ± 2.17	0.086
Basal LH (MIU/mL)	4.39 ± 3.52	4.86 ± 4.34	0.086
Basal E2 (mmol/L)	146.36 ± 62.25	160.94 ± 85.98	0.004
AFC	15.84 ± 5.95	14.92 ± 5.44	0.079
Semen analysis			
Semen density (Million/mL)	47.79 ± 49.19	46.87 ± 47.7	0.778
Sperm motility rate (%)	26.13 ± 21.44	25.10 ± 20.42	0.469
IVF-ET indications			0.465
Tubal factor	131 (31.41)	138 (30.60)	
Endometriosis	18 (4.32)	11 (2.44)	
Ovulation disorder	75 (17.99)	93 (20.62)	
Male factor	130 (31.18)	132 (29.27)	
Recurrent miscarriage	5 (1.20)	8 (1.77)	
Diminished ovarian reserve	2 (0.48)	0 (0.00)	
Unexplained infertility	51 (12.23)	60 (13.30)	
Chromosomal abnormalities	5 (1.20)	9 (2.00)	
History of IVF failure	87 (20.86)	82 (18.18)	0.319
Ovulation hyperstimulation protocol			0.994
Long GnRH agonist	150 (35.97)	163 (36.14)	
Ultralong GnRH agonist	61 (14.63)	68 (15.08)	
GnRH antagonist	202 (48.44)	216 (47.89)	
Others	4 (0.96)	4 (0.89)	
Trigger medication			1.000
HCG/r-HCG	357 (85.61)	385 (85.37)	
HCG/r-HCG + GnRH agonist	60 (14.39)	65 (14.41)	
GnRH agonist	0 (0.00)	1 (0.22)	
Duration of Gn applied (days)	11.05 ± 2.07	11.11 ± 2.12	0.658
Total Gn applied (units)	1963.17 ± 863.52	2001.81 ± 792.31	0.492
LH on the day of hCG (mIU/mL)	1.65 ± 1.90	1.72 ± 2.61	0.660
E2 on the day of hCG (mmol/L)	15,789.18 ± 10,758.43	13,284.72 ± 7575.05	<0.001
Progesterone on the day of hCG (pmol/L)	3.09 ± 1.86	2.78 ± 1.61	0.011
Total oocytes retrieved	21.86 ± 11.85	20.24 ± 10.22	0.031
2PN rate (%)	12.56 ± 7.38	12.14 ± 6.90	0.398
Number of cleavage-stage embryos	14.97 ± 8.88	14.18 ± 7.99	0.167
Type of fertilization			0.085
IVF	214 (51.31)	231 (51.22)	
ICSI	181 (43.41)	206 (45.68)	
Half-ICSI	21 (5.04)	10 (2.22)	
Cryopreservation	1 (0.24)	4 (0.89)	

Data are presented as n (%) and mean ± standard deviation. AFC: antral follicle count; AMH: anti-müllerian hormone; BMI: body mass index; E2: estradiol; FSH: follicle-stimulating hormone; Gn: gonadotropin; GnRH: gonadotropin-releasing hormone; HCG: human chorionic gonadotropin; ICSI: intracytoplasmic sperm injection; IVF: in vitro fertilization; IVF-ET: in vitro fertilization and embryo transfer; LH: luteinizing hormone; 2PN: 2 pronuclei (the presence of two clearly distinct pronuclei and two polar bodies); r-HCG: recombinant human chorionic gonadotropin.

**Table 2:** Clinical features and treatment of OHSS of the participants with or without hydrothorax.

	Ascites Alone Group (n = 417)	Ascites Combined with Hydrothorax Group (n = 451)	*p*
Time of symptom onset (days after trigger)	10.57 ± 5.44	11.96 ± 5.61	<0.001
Type of OHSS			0.994
Early onset	207 (49.64)	224 (49.67)	
Late onset	210 (50.36)	227 (50.33)	
Readmission rate	10 (2.40)	8 (1.77)	0.519
Hospital stay (days)	4.13 ± 2.83	2.79 ± 3.02	<0.001
Weight gain (kg)	2.02 ± 3.09	1.68 ± 4.35	0.179
Abdominal symptoms			<0.001
Asymptomatic *	5 (1.20)	44 (9.76)	
Bloating	365 (87.53)	384 (85.14)	
Abdominal pain *	47 (11.27)	23 (5.10)	
Chest symptoms *			<0.001
Asymptomatic	346 (82.97)	181 (40.13)	
Chest tightness and shortness of breath	70 (16.79)	259 (57.43)	
Difficulty breathing	1 (0.24)	11 (2.44)	
Severity of ascites *			<0.001
Mild	28 (6.71)	10 (2.22)	
Moderate	56 (13.43)	180 (39.91)	
Massive	333 (79.86)	261 (57.87)	
Leukocyte (×10^9^/L)	16.06 ± 5.07	14.24 ± 5.11	<0.001
HCT	0.44 ± 0.05	0.42 ± 0.05	<0.001
PLT (×10^9^/L)	349.31 ± 89.52	337.69 ± 88.14	0.055
Urine specific gravity	1.03 ± 0.01	1.03 ± 0.02	0.012
D-Dimer (mg/L)	1.03 ± 0.81	1.1 ± 0.81	0.212
ALT (U/L)	52.63 ± 55.33	53.68 ± 51.40	0.793
AST (U/L)	45.92 ± 41.19	46.49 ± 35.52	0.845
TP (g/L)	57.22 ± 11.24	59.04 ± 10.60	0.030
ALB (g/L)	30.12 ± 6.91	31.29 ± 6.84	0.026
K^+^ (mmol/L)	3.96 ± 0.41	3.91 ± 0.39	0.085
Na^+^ (mmol/L)	134.34 ± 3.84	134.60 ± 3.95	0.380
Cr (umol/L)	55.16 ± 10.52	55.46 ± 10.63	0.698
Bilateral ovarian mean (cm)	8.15 ± 1.55	7.93 ± 1.58	0.040
Albumin infusion treatment	23 (5.52)	18 (3.99)	0.290
Anticoagulation treatment	133 (31.89)	140 (31.04)	0.787
Peritoneal drainage treatment	212 (50.84)	164 (36.36)	<0.001
Peritoneal drainage treatment duration (days)	1.16 ± 1.75	0.96 ± 1.75	0.085
Total volume of peritoneal drainage (mL)	2494.19 ± 3501.80	1943.8 ± 3477.63	0.020
Pleural drainage treatment	0 (0.00)	12 (2.66)	<0.001
Pleural drainage treatment duration (days)	0 ± 0	0.03 ± 0.16	<0.001
Total volume of pleural drainage (mL)	0 ± 0	21.55 ± 133.88	0.001

Data are presented as n (%) and mean ± standard deviation. * Means statistically significant was found between two groups. ALB: albumin; ALT: alanine aminotransferase; AST: glutamic-oxalacetic transaminase; Cr: creatinine; HCT: hematokrit; K+: kalium; Na+: sodion; PLT: platelets; TP: total protein.

**Table 3:** Embryology outcomes and clinical reproductive outcomes of the participants with or without hydrothorax.

	Ascites Alone Group (n = 210)	Ascites Combined with Hydrothorax Group (n = 227)	*p*
Number of embryos transferred	1.92 ± 0.27	1.93 ± 0.26	0.959
Type of embryos transferred			0.851
Cleavage embryo	199 (94.76)	216 (95.15)	
Blastocyst	11 (5.24)	11 (4.85)	
Live birth rate	167 (79.52)	199 (87.67)	0.021
Miscarriage rate	30 (14.29)	15 (6.61)	0.008
Early miscarriage rate	16 (7.62)	12 (5.29)	0.320
Mid-term miscarriage rate	14 (6.67)	3 (1.32)	0.004
Surgical foetal reduction	13 (6.19)	16 (7.05)	0.719
Natural foetal reduction	12 (5.71)	19 (8.37)	0.280
Full-term birth	132 (79.04)	149 (74.87)	0.347
Twin pregnancy	61 (36.53)	79 (39.70)	0.534
Pregnancy complications	12 (6.09)	18 (8.41)	0.366
Gestational week at delivery (week)	37.66 ± 2.29	37.36 ± 2.42	0.222
Average weight at birth(kg)	2917.62 ± 583.07	2851.25 ± 575.79	0.276

Data are presented as n (%) and mean ± standard deviation.

**Table 4:** Multivariate logistic regression analysis of factors for live birth.

	Combined with Hydrothorax		Volume of Hydrothorax	
	OR	*p*	OR	*p*
Time of symptom onset (days after trigger)	0.857 (0.795, 0.925)	<0.001	0.862 (0.798, 0.931)	<0.001
Foetal reduction		0.011		0.022
Yes	1		1	
No	13.796 (1.808, 105.288)		10.835 (1.416, 82.891)	
History of IVF failure		<0.001		<0.001
Yes	1		1	
No	0.338 (0.184, 0.622)		0.312 (0.167, 0.583)	
Number of oocytes retrieved	1.102 (1.015, 1.196)	0.021	1.110 (1.021, 1.206)	0.014
Number of embryos transferred	0.248 (0.077, 0.800)	0.020	0.251 (0.077, 0.819)	0.022
Type of embryos transferred		0.014		0.007
Cleavage embryo	1		1	
Blastocyst	0.123 (0.023, 0.654)		0.099 (0.018, 0.534)	
Abdominal symptoms		0.070		0.067
None	1		1	
Bloating	0.821 (0.189, 3.558)		0.792 (0.155, 4.040)	
Abdominal pain	2.658 (0.475, 14.872)		2.601 (0.397, 17.053)	

Adjusting for potential confounders including age, BMI, the number of embryos transferred, the type of embryos transferred, and variables with *p* < 0.1 in univariate analysis (previous IVF failure, total number of oocytes retrieved, time of symptom onset, abdominal symptoms, history of foetal reduction, Gn dosage, and WBC).

## Data Availability

The datasets used and/or analysed during the current study are available from the corresponding author upon reasonable request.

## Supplementary Materials

The following supporting information can be downloaded at: https://www.mdpi.com/article/10.3390/jcm12196210/s1, Table S1: Univariate analysis of the factors for live birth.
