# Supplementary material for: Clinical Features of Severe Ovarian Hyperstimulation Syndrome with Hydrothorax
Source: J Clin Med. 2023 Sep 26;12(19):6210. doi: 10.3390/jcm12196210 (PMC10573803; doi:10.3390/jcm12196210)
Supplement: Supplementary file 1 [file jcm-12-06210-s001.zip › jcm-2552779-supplementary.pdf]

Supplemental Table S1：Univariate analysis of the factors for live birth

	Live birth group(N=366)	Non-live birth group(N=71)	P
Age	31.17±3.51	31.24±3.95	0.876
BMI（kg/㎡）	22.34±3.33	22.89±3.36	0.197
Duration of infertility (years)	3.39±2.39	3.35±2.48	0.912
Gravidity (times)	0.52±0.85	0.48±0.71	0.709
Delivery (times)	0.05±0.25	0.03±0.17	0.436
Basal FSH (MIU/mL)	5.76±2.19	6.00±2.01	0.385
Basal LH (MIU/mL)	4.35±3.57	4.39±3.24	0.940
Basal E2 (mmol/L)	151.45±72.54	143.06±49.22	0.230
AFC	14.14±5.36	15.20±5.70	0.183
Semen analysis			
Semen density(Million/ml)	48.72±49.26	49.43±52.34	0.912
Sperm motility rate(%)	25.22±20.92	27.75±22.39	0.356
IVF-ET indications			0.988
Tubal factor	118(32.24)	26(36.62)	
Endometriosis	14(3.83）	2（2.82）	
Ovulation disorder	67（18.31）	12（16.90）	
Male factor	116（31.69）	22（30.99）	
Recurrent miscarriage	49(1.09)	1(1.41)	
Diminished ovarian reserve	2(0.55)	0(0.00)	
Unexplained infertility	45(12.30)	8(11.27)	
History of IVF failure	65(17.76)	30(42.25)	＜0.001
Ovulation hyperstimulation protocol			0.266
Long protocol	122（33.33）	17（23.94）	
Extra-long protocol	62（16.94）	12（16.90）	
Antagonist protocol	180（49.18）	41（57.75）	
Others	2（0.55）	1（1.41）	
Trigger medication			0.530
HCG/r-HCG	338（92.35）	64（90.14）	
HCG/r-HCG+GnRH agonist	28（7.65）	7（9.86）	
Duration of Gn applied (days)	11.17±2.30	11.23±2.02	0.841
Total Gn applied (units)	2070.56±826.11	2291.90±1168.75	0.056
LH on the day of hCG (mIU/mL)	1.72±2.30	1.67±2.16	0.862
E2 on the day of hCG (mmol/L)	9128.22±3771.61	9266.42±4004.52	0.780
Progesterone on the day of hCG (pmol/L)	2.24±1.06	2.29±1.17	0.711
Total oocytes retrieved	13.20±3.90	14.66±3.29	0.003
2PN rate(%)	8.37±3.37	8.69±3.15	0.458
Number of cleavage-stage embryos	9.80±3.74	10.10±3.53	0.532
Type of fertilization			0.685
IVF	210(57.38)	45(63.38)	
ICSI	150(40.98)	25(35.21)	
Half-ICSI	6(1.64)	1(1.41)	
Time of symptom onset (days after trigger)	16.28±3.77	13.51±4.74	＜0.001
Readmission rate	14(3.83)	2(2.82)	1.000
Hospital stay (days)	4.65±3.41	4.61±3.19	0.914
Weight gain (kg)	2.57±3.45	2.15±4.28	0.366
Abdominal symptoms			0.044
Bloating	334(91.26)[a]	59(83.10)[b]	
Abdominal pain	18(4.92)[a]	9(12.68)[b]	
Asymptomatic	14(3.83)	3(4.23)	
Chest symptoms			0.948
Chest tightness and shortness of breath	134(36.61)	27(38.03)	
Difficulty breathing	4(1.09)	0(0.00)	
Asymptomatic	228(62.30)	44(61.97)	
Severity of ascites			0.544
Mild	8(2.19)	2(2.82)	
Moderate	67(18.31)	9(17.68)	
Massive	291(79.51)	60(84.51)	

Leukocyte ($\times10^9$ /L)	14.61±5.00	16.42±7.50	0.054
HCT	0.43±0.05	0.43±0.05	0.522
PLT ($\times10^9$ /L)	368.64±92.57	358.96±80.29	0.411
Urine specific gravity	1.03±0.01	1.03±0.01	0.347
D-Dimer (mg/L)	0.81±0.57	0.90±0.51	0.218
ALT (U/L)	68.05±62.91	67.23±61.43	0.922
AST (U/L)	55.04±45.16	55.33±44.57	0.963
TP (g/L)	55.92±10.20	56.89±11.37	0.498
ALB (g/L)	29.50±6.59	30.00±6.76	0.577
K^+ (mmol/L)	3.89±0.36	3.85±0.36	0.344
Na^+ (mmol/L)	134.23±4.06	133.78±5.28	0.448
Cr (umol/L)	52.59±10.10	54.59±9.78	0.142
Bilateral ovarian mean(cm)	7.38±1.37	7.53±1.23	0.397
Albumin infusion treatment	26（7.10）	4（5.63）	0.801
Anticoagulation treatment	99（27.05）	26（36.62）	1.102
Peritoneal drainage treatment	188（51.37）	39（54.93）	0.582
Peritoneal drainage treatment duration (days)	1.59±2.17	1.76±2.19	0.545
Total volume of peritoneal drainage (ml)	3159.17±4344.34	3440.92±4106.98	0.614
Pleural drainage treatment	6（1.64）	0（0.00）	0.595
Pleural drainage treatment duration (days)	0.02±0.13	0±0	0.014
Total volume of pleural drainage (ml)	13.55±109.56	0±0	0.018
Number of embryos transferred	1.93±0.25	1.89±0.32	0.271
Type of embryos transferred			1
Cleavage embryo	347(94.81)	68(95.77)	
Blastocyst	19(5.19)	3(4.23)	
Surgical foetal reduction	29（7.92）	0（0.00）	0.008
Natural foetal reduction	30（8.20）	1(1.41)	0.041
Total foetal reduction	59（16.12）	1(1.41)	0.001
Ascites alone or combined with hydrothorax			0.021
Ascites alone	167(45.63)	43(60.56)	
Ascites combined with hydrothorax	199(54.37)	28(39.44)	
Distribution of hydrothorax			0.057
Unilateral	71(19.40)	12(16.90)	
Bilateral	128(34.97)[a]	16(22.54)[b]	
None	167(45.63)[a]	43(60.56)[b]	
Volume of hydrothorax			0.006
Mild	88（24.04）[a]	9(12.68)[b]	
Moderate	87(23.77)	19(26.76)	
Massive	24(6.56)[a]	0(0.00)[b]	
None	167(45.63)[a]	43(60.56)[b]	
Location of hydrothorax			0.124
Isolated right side	58(15.85)	10(14.08)	
Isolated left side	13(3.55)	2(2.82)	
Bilateral	128(34.97)[a]	16(22.54)[b]	
None	167(45.63)[a]	43(60.56)[b]	
